# Investigating academic performance and perceptions of human physiology and exercise physiology courses among undergraduate students of physical education at a Brazilian public university

**DOI:** 10.1186/s12909-024-05058-1

**Published:** 2024-01-24

**Authors:** Mila Alves Matos Rodrigues, Rizia Rocha Silva, Douglas Assis Teles Santos, João Victor Rosa de Freitas, Paulo Gentil, Carlos Alexandre Vieira, Ana Cristina Silva Rebelo, Marília Santos Andrade, Mário Hebling Campos, Gustavo de Conti Teixeira Costa, Beat Knechtle, Rodrigo Luiz Vancini, Claudio Andre Barbosa de Lira

**Affiliations:** 1 Faculdade de Educação Física e Dança, Universidade Federal de Goiás, Goiânia, Brazil; 2 Universidade do Estado da Bahia, Campus X, Teixeira de Freitas, Bahia, Brazil; 3 Instituto de Ciências Biológicas, Universidade Federal de Goiás, Goiânia, Brazil; 4 Departamento de Fisiologia, Universidade Federal de São Paulo, São Paulo, Brazil; 5 Institute of Primary Care, University of Zurich, 8006 Zurich, Switzerland; 6 Medbase St. Gallen Am, Vadianplatz, 9000 St., Gallen, Switzerland; 7 Centro de Educação Física e Desportos, Universidade Federal do Espírito Santo, Vitória, Brazil; 8 Avenida Esperança, s/n, Conjunto Itatiaia, CEP 74690-900 Goiânia, Goiás Brazil

**Keywords:** Learning, Physiology, Professional practice, Students, Teaching, University

## Abstract

**Purpose:**

To compare the academic performance of undergraduate students in physical education who studied exercise physiology before and after studying human physiology and investigate students’ perceptions of human physiology and exercise physiology courses.

**Methods:**

This study included 311 undergraduate students pursuing a bachelor’s degree in physical education. Participants were divided into two groups: those who had previously attended and completed the human physiology course (group 1, *n* = 212, 68.2%) and those who had not previously attended or had attended but failed the human physiology course (group 2, *n* = 99, 31.8%). The prevalence ratio (PR) and 95% confidence interval (95% CI) were calculated using a Poisson regression model with a robust variance estimator. The second aim comprised 67 students with bachelor’s degrees in physical education who completed an electronic questionnaire about their perceptions of human physiology and exercise physiology curriculum.

**Results:**

Compared with those who attended human physiology and passed, those who had not previously attended or had attended but failed the human physiology course have a higher PR of 2.37 (95% CI, 1.68–3.34) for failing exercise physiology. Regarding the students’ perceptions of human physiology and exercise physiology courses, most students reported that they were challenging (58.2% and 64.2%, respectively), but they also recognized the importance of these courses for professional practice (59.7% and 85.1%, respectively).

**Conclusion:**

Human physiology should be considered a prerequisite for an undergraduate course leading to a bachelor’s degree in physical education. Furthermore, students considered human physiology and exercise physiology courses important yet challenging. Therefore, continuous student assessment is vital for improving the teaching–learning process.

## Introduction

Anatomy, biochemistry, and physiology are studied in the first year of health undergraduate courses and considered basic sciences necessary for understanding later years’ curriculum [1]. Human physiology is part of the fundamental course cycle of a bachelor’s degree in physical education. It aims to teach students about physiological system functions and mechanisms that affect the internal environment, such as body temperature, blood glucose, and arterial oxygen content [1]. For these reasons, the human physiology course may serve as the foundation for understanding exercise physiology.

Exercise physiology courses have traditionally taught exercise’s acute and chronic effects and the physiological mechanisms responsible for these effects [2]. Additionally, exercise physiology can explore aspects of exercise performed in specific environments and conditions (e.g., heat, cold, and high altitude) and populations (e.g., persons with chronic noncommunicable diseases, children, women, and the elderly) [3]. Therefore, this course aims to understand how physiological systems respond to exercise and how environmental and individual conditions can change these physiological systems and their response to physical exercise [1]. Furthermore, students should be able to select activities that contribute to the maintenance and/or acquisition of a healthy and active lifestyle while also improving exercise performance and physical conditioning at the end of the exercise physiology course [1]. Therefore, the course should help students improve their ability to apply their understanding of exercise physiology in areas such as physical activity for health and sports performance [4].

Despite the importance of human physiology courses, research on student and professor perceptions of physiology consistently presents it as challenging [5–7]. Students consider the physiology course to be complex and time-consuming [6]. Professors often point out that the problem is not due to teaching methods but rather due to learning factors, such as students confusing memorizing with learning and thinking about physiological systems separately rather than linking them [5]. As a result, many students and health professionals have significant misconceptions about physiology, particularly cardiovascular [8], respiratory [9, 10], and exercise physiology [11]. Therefore, studies on strategies to reduce misconceptions and other aspects of problematic learning are necessary.

A thorough understanding of human physiology and exercise physiology is most likely necessary. However, some undergraduate courses lack prerequisite or co-requisite characteristics in their curricular structures [12], which can result in significant gaps in student formation and impede future professional growth.

There are no regulations governing the order of human physiology and exercise physiology courses in Brazilian bachelor’s degrees in physical education. It is not mandatory by current regulations, but it demonstrates that exercise physiology is in the common stage. Consequently, many undergraduate courses enable students to choose the order of the content. Because of this autonomy, the student can take an exercise physiology course before completing a human physiology course. Such factors may have a negative impact on a student’s academic performance in exercise physiology. Therefore, studies that objectively investigate this issue are necessary, and the findings may provide a curriculum guideline for undergraduate physical education courses.

Another interesting topic is evaluating students’ perceptions of a particular subject. Most studies that examined students’ perceptions, importance, and performance were conducted in anatomy courses [13–16], and little is known about students’ perceptions of exercise physiology. Therefore, a study to analyze this gap is needed.

Furthermore, a study into this topic is necessary because some prerequisite courses are better used at the appropriate time. Examining the academic performance of undergraduate physical education bachelor students is an interesting and objective approach to reducing this gap.

Therefore, the present study aimed to compare the academic performance of physical education undergraduate students who studied exercise physiology before studying human physiology with those who studied exercise physiology after studying human physiology. Second, we evaluated students with bachelor’s degrees in physical education’s perceptions of human physiology and exercise physiology courses. We hypothesized that those who did not study human physiology would perform lower academically in exercise physiology.

## Materials and methods

### Study design and participants

To answer our purposes, two studies were conducted. The first was a descriptive retrospective study of 311 students (192 men and 119 women) with bachelor’s degree in physical education at the Faculty of Physical Education and Dance of the Federal University of Goiás (FEFD-UFG, Goiânia, Brazil). This aim was to compare the academic performance of physical education undergraduate students who studied exercise physiology before human physiology with those who studied exercise physiology after human physiology.

The second study was a cross-sectional study of 67 students (47 men and 20 women) developed to assess the perceptions of bachelor’s degree in physical education students on human physiology and exercise physiology courses. The student must take an exercise physiology course to be included in the study. All study procedures were approved by the Research Ethics Committee of the Federal University of Goiás (protocol CAAE number 54808121.7.0000.5083).

### Study procedures

The UFG Dean’s Office for Undergraduate Studies provided the students’ school records from 2009 to 2022, from which the following data were extracted: final grades in human physiology and exercise physiology and the order in which the coursework was taken for the first study. Subsequently, the students were divided into two groups depending on their performance in human physiology: group 1 (students who studied human physiology and passed) and group 2 (students who had not previously attended or had attended but failed in human physiology).

To conduct the study’s second aim, students enrolled in the exercise physiology course in 2021 and 2022 were given an electronic questionnaire generated on Google Forms (Google Corp., USA). The questionnaire used in this study was designed to keep participants anonymous, and respondents were not required to disclose any personal information. This anonymity was implemented to encourage participants to provide open and honest comments. Participants in our sample included those who did not take the human physiology course and those who did. Students that did not take the human physiology course evaluated the human physiology course based on their general knowledge of the subject, while students that took the human physiology course evaluated the course after participating in the human physiology course. Furthermore, the questionnaire was given to students after they received their grades for the human physiology course.

This questionnaire had four multiple-choice questions using a Likert scale (4- or 5-point scale) or dichotomous (yes/no) answers to assess students’ perceptions of human physiology and exercise physiology courses. The questionnaire specifically collected data on human physiology and exercise physiology students’ perceptions of course level (1 = *extremely difficult*, 2 = *difficult*, 3 = *regular*, 4 = *easy*, and 5 = *extremely easy*), importance of professional performance (1 = *extremely unimportant*, 2 = *unimportant*, 3 = *indifferent*, 4 = *important*, and 5 = *extremely important*), workload (1 = *extremely inadequate*, 2 = *inadequate*, 3 = *adequate*, and 4 = *extremely adequate*), and interest in optional courses (1 = *yes* and 2 = *no*). Students were invited to participate in the study during the second week of class. The access link was available for 1 week. 0.25 points were added to the final grade as an incentive for participation.

The method used the repeated application of the instrument to the same participants with a 7-day interval between applications to evaluate the reproducibility of the produced questionnaire. Based on the questionnaire results, two types of reliability were established: stability over time (*κ* concordance test) and internal consistency (Cronbach’s *α*). For Likert scale responses (weighted *κ*: 0.315 to 0.636; *p* < 0.01) and dichotomous responses (Cohen’s *κ*: 0.374 and 0.745; *p* < 0.01), the results varied from fair to significant. Internal consistency ranged from moderate to high results (test *α*: 0.628–0.802; retest *α*: 0.222–0.733).

### Characteristics of the evaluated human physiology, exercise physiology, and undergraduate physical education courses at the Federal University of Goiás

The human physiology course had an 80-h schedule (64 theoretical hours and 16 practical hours), and according to the flow provided by the undergraduate course pedagogical project (PPC), it should be taken by the student in the third semester of the undergraduate course. The study of physiological systems is emphasized in the human physiology course, with the following contents: cellular biophysics, muscular, nervous, cardiovascular, respiratory, digestive, renal, and endocrine systems.

The exercise physiology course had a schedule of 64 h (48 theoretical hours and 16 practical hours), and according to the PPC’s proposed flow, it should be taken by the student in the fourth semester of the course. The exercise physiology course aims to teach the physiological mechanisms that occur in the human body as a result of physical exercise (acute or chronic) on the following physiological systems: muscular, nervous, cardiovascular, respiratory, digestive, renal, and endocrine systems, as well as temperature regulation, acid–base balance, and metabolism during physical activity.

As an indicator of academic performance, the grading system utilized in UFG is based on a scale of 0 to 10, with 10 maximum grade and a passing grade is 6.0.

Due to the lack of prerequisites, the PPC for the physical education undergraduate course at UFG does not impose a follow-up order on the student. Thus, the student can design the course sequence. All professors responsible for teaching human physiology and exercise physiology have a doctorate and are involved in research activities.

### Statistical analyses

The Statistical Package for the Social Sciences (version 26.0, IBM Corp., USA) was used for analysis. Descriptive analyses are presented as relative and/or absolute frequencies and median (interquartile range). Poisson regression using generalized linear models with a robust estimator, adjusted by frequency in class in a multivariate model, was used to assess the prevalence ratios of performance in human physiology and exercise physiology. A 5% significance level and a 95% CI were used.

## Results

In total, 311 undergraduate students in physical education took exercise physiology courses; 212 (68.2%) students passed, and 99 (31.8%) failed in human physiology; 230 (73.9%) students passed, and 81 (26.1%) failed in exercise physiology. The final grade in exercise physiology course for those who completed the human physiology course (*n* = 212) was 6.9 (1.8; data are expressed as median [interquartile range]), and the final grade for those who failed the human physiology course (*n* = 99) was 5.5 (2.7).

Human physiology and exercise physiology coursework were evaluated as difficult by 58.2% and 64.2% of students’ perceptions, respectively, and 59.7% and 85.1% were considered highly important for professional practice, respectively (Table 1).

**Table 1:** Students’ perceptions of physical education undergraduate students in human physiology and exercise physiology coursework

Item	Human physiology (*n* = 67)	Exercise physiology (*n* = 67)
How do you evaluate the level of the coursework?		
Extremely hard	4 (6.0%)	6 (9.0%)
Difficult	39 (58.2%)	43 (64.2%)
Regular	24 (35.8%)	17 (25.4%)
Easy	0 (0.0%)	1 (1.5%)
Extremely easy	0 (0.0%)	0 (0.0%)
How do you evaluate the coursework for your future professional performance?		
Extremely unimportant	3 (4.5%)	1 (1.5%)
Unimportant	0 (0.0%)	0 (0.0%)
Indifferent	1 (1.5%)	0 (0.0%)
Important	23 (34.3%)	9 (13.4%)
Extremely important	40 (59.7%)	57 (85.1%)
How do you evaluate the course load?		
Extremely inappropriate	13 (19.4%)	3 (4.5%)
Inadequate	0 (0.0%)	15 (22.4%)
Proper	49 (73.1%)	40 (59.7%)
Extremely suitable	5 (7.5%)	9 (13.4%)
If UFG offered it, you would take the following electives:		
Yes	53 (79.1%)	63 (94.0%)
No	14 (20.9%)	4 (6.0%)

Note. Data of the first application (test)

As shown in Table 2, we found that students who had not previously attended or had attended but failed human physiology coursework had a higher prevalence ratio (PR) of 2.37 (95% CI, 1.68–3.34) of failing exercise physiology coursework when compared with those who had done human physiology and passed it.

**Table 2:** Poisson regression models for not passed in human physiology and exercise physiology

Variables	Not passed in human physiology			
	Crude	*p*	Multivariate^a^	*p*
	PR (95% CI)		PR (95% CI)	
Exercise physiology				
Not passed (group 2)	2.46 (1.82–3.33)	**< 0.001**	2.37 (1.68–3.34)	**< 0.001**
Passed (group 1)	1		1	

Note. 95% CI, 95% confidence interval; PR, prevalence ratio

^a^Adjusted by frequency in the class

## Discussion

The primary aim of this study was to compare the academic performance of physical education undergraduate students who studied exercise physiology before human physiology with those who studied exercise physiology after human physiology. Second, we investigated students’ perceptions with bachelor’s degrees in physical education on human physiology and exercise physiology coursework. The main findings were as follows: (a) students who previously did not attend or failed human physiology coursework are more likely to fail exercise physiology coursework, and (b) students classified human physiology and exercise physiology as complex, but they recognized the importance of both courses for professional practice.

Previous studies assessed students’ perceptions of human physiology and anatomy courses [5–7, 17], demonstrating that students consider human physiology and anatomy difficult; however, to the best of our knowledge, no studies have evaluated students’ perceptions of exercise physiology coursework. Despite the apparent difficulties, students overwhelmingly recognized the significance of human and exercise physiology for their future professional endeavors, according to our findings. Therefore, the findings are consistent with the present literature because students here classified exercise physiology as complex, as previous studies for human anatomy and physiology revealed.

Many sources of difficulty in studying physiology have been identified in previous research, including intrinsic difficulty in teaching [5, 7, 18, 19], motivational component [19], time commitment [7, 19], large amount of information [5, 18], little time for studying outside of the classroom, lack of prior knowledge, and intrinsic difficulty in coursework [18].

Slominski et al. [17] found that causal reasoning ability, systems thinking ability, and students’ view that memorization is the same as learning are the factors listed as barriers to students’ learning. Although these aspects were not analyzed in the present study, it is plausible to assume that a similar situation may occur due to the same nature of the course in the present study.

Another particular finding from this study is that those who did not attend or failed human physiology have a higher prevalence ratio of failing exercise physiology course. This incontrovertible data indicates that students should pass the human physiology course before enrolling in exercise physiology. Previous studies have shown that although studying physiology necessitates a solid understanding of basic sciences, students often lack this basis [5, 7, 20]. Michael [5] conducted a study after noting that a lack of prior knowledge may explain students’ conceptual difficulties. Nasre-Nasser et al. [7] reported a similar result, finding that 68.8% of professors claimed that it is common for students to be enrolled in physiology without having the necessary knowledge of basic sciences and prerequisite skills. Such findings highlight the indisputable importance of prior knowledge for exemplary performance in physiology, which may explain why students who previously attended the human physiology course performed better academically than those who did not participate or who attended but failed the exercise physiology course. Therefore, students must be introduced to basic information before being exposed to more particular and professional data in the field. This issue becomes more pressing when students fail to recognize the importance of prior knowledge and, therefore, fail to identify a lack of it as a source of their difficulties [5]. In this context, the results reported here can be used to objectively advise educational institution faculty staff in developing the order of curricular contents (at least for the undergraduate physical education course).

This study has some limitations. The grouping strategy may have resulted in a bias in which students in group 1 performed best (passed) and students in group 2 performed worst (failed). Regarding the results about perception, some participants did not take the human physiology course, while others did. Therefore, the students evaluated here did not present the same experience with the human physiology course. Furthermore, the perception provided by students who had taken the human physiology course before completing the questionnaire may be affected (consciously or unconsciously) by their grades. Nevertheless, we believe that these limitations do not preclude the results of the study from being derived.

## Conclusion

Students who did not attend or fail human physiology courses are likelier to fail exercise physiology coursework. Human physiology should be considered a prerequisite discipline in a bachelor’s degree in physical education course. Continuous assessment of students and professors’ perceptions, needs, and difficulties is critical for improving the teaching–learning process. It is essential to emphasize the importance of understanding human physiology as a prerequisite for studying exercise physiology.

## Data Availability

The datasets generated and/or analyzed during the present study are not publicly available because they are data from the Federal University of Goiás. However, they are available upon reasonable request from the corresponding author.
